# MicroRNA breed and parent-of-origin effects provide insights into biological pathways differentiating cattle subspecies in fetal liver

**DOI:** 10.3389/fgene.2023.1329939

**Published:** 2023-12-13

**Authors:** Callum MacPhillamy, Yan Ren, Tong Chen, Stefan Hiendleder, Wai Yee Low

**Affiliations:** ^1^ The Davies Research Centre, School of Animal and Veterinary Sciences, University of Adelaide, Roseworthy, SA, Australia; ^2^ Robinson Research Institute, The University of Adelaide, North Adelaide, SA, Australia

**Keywords:** mRNA-miRNA, differentially expressed micrornas (DE-miRNAs), differentially expressed genes (DEGs), Brahman, Angus, fetal liver

## Abstract

**Introduction:** MicroRNAs (miRNAs) play a crucial role in regulating gene expression during key developmental processes, including fetal development. Brahman (*Bos taurus indicus*) and Angus (*Bos taurus taurus*) cattle breeds represent two major cattle subspecies with strikingly different phenotypes.

**Methods:** We analyzed miRNA expression in liver samples of purebred and reciprocal crosses of Angus and Brahman to investigate breed and parent-of-origin effects at the onset of accelerated fetal growth.

**Results:** We identified eight novel miRNAs in fetal liver samples and 14 differentially expressed miRNAs (DEMs) between purebred samples. Correlation of gene expression modules and miRNAs by breed and parent-of-origin effects revealed an enrichment of genes associated with breed-specific differences in traits such as heat tolerance (Brahman) and fat deposition (Angus). We demonstrate that genes predicted to be targets of DEMs were more likely to be differentially expressed than non-targets (*p*-value < 0.05). We identified several miRNAs (bta-miR-187, bta-miR-216b, bta-miR-2284c, bta-miR-2285c, bta-miR-2285cp, bta-miR-2419-3p, bta-miR-2419-5p, and bta-miR-11984) that showed similar correlation patterns as bta-miR-2355-3p, which has been associated with the glutamatergic synapse pathway, a key facilitator of heat tolerance. Furthermore, we report Angus-breed-specific miRNAs (bta-miR-2313-5p, btamiR-490, bta-miR-2316, and bta-miR-11990) that may be involved in fat deposition. Finally, we showed that the DEMs identified in fetal liver are involved in Rap1, MAPK, and Ras signalling pathways, which are important for fetal development, muscle development and metabolic traits such as fat metabolism.

**Conclusion:** Our work sheds light on the miRNA expression patterns that contribute to gene expression differences driving phenotypic differences in indicine and taurine cattle.

## Introduction

The two main lineages of modern cattle breeds are derived from two separate domestications of the wild auroch (*Bos primigenius*) (McTavish et al., 2013). The first domestication event occurred in the Fertile Crescent ∼10,000 years ago and gave rise to *Bos taurus taurus* (Bruford et al., 2003; Ajmone-Marsan et al., 2010; MacHugh et al., 2017). A second domestication from a different auroch subspecies occurred ∼1,500 years later in the Indus Valley (Loftus et al., 1994) and gave rise to *Bos taurus indicus*. The domesticated subspecies are referred to as taurine and indicine cattle; the Angus breed is a representative of taurine cattle, and the Brahman breed of indicine cattle (McTavish et al., 2013). The two breeds have starkly contrasting phenotypes; e.g., Angus cattle have been selected for growth traits and meat production (Elzo et al., 2012) and hence have shorter gestation length and mature earlier than Brahman cattle with a muscle fibre composition conducive to meat tenderness (Casas et al., 2011; Xiang et al., 2013). Brahman cattle, in contrast, have superior heat and disease tolerance traits and are thus adapted to tropical environments, but they mature more slowly and have coarser muscle fibres and tougher meat (Chase et al., 1997; Elzo et al., 2012; Dikmen et al., 2018; Goszczynski et al., 2018).

Although Angus and Brahman cattle differ by ∼1% genetically at the nucleotide level and have a different Y chromosome structure (Goldammer et al., 1997; Low et al., 2020), they produce fertile inter-subspecific hybrids (Hiendleder et al., 2008) that have enabled the investigation of genetic and epigenetic factors involved in phenotypic differences of quantitative traits (Hiendleder et al., 2008; Mao et al., 2008; Xiang et al., 2013; Xiang et al., 2014; Akanno et al., 2018; Gobena et al., 2018; Andrade et al., 2022). Genetic and epigenetic regulatory changes that underlie the phenotypic differences between taurine and indicine cattle are of substantial interest from both scientific and economic perspectives, i.e., understanding complex trait biology and using this knowledge to combine desirable traits from both subspecies (Li et al., 2023).

Differences in the expression of microRNA (miRNA), small non-coding RNAs of ∼22 bp essential for epigenetic regulation of gene expression (Eulalio et al., 2008), could be one of the main drivers of extensive transcriptomic and phenotypic differences observed between taurine and indicine cattle (Liu et al., 2021). However, little is known about miRNAs that differentiate the subspecies (Hanif et al., 2018; Dong et al., 2023). MiRNAs have been implicated in a wide range of biological processes, including growth and development (Awamleh et al., 2019; Morales-Roselló et al., 2022), cell differentiation (Galagali and Kim, 2020), metabolism (Rottiers and Näär, 2012) and response to environmental stimuli (Vrijens et al., 2015; Liu et al., 2020). In general, miRNAs are highly conserved across species (Macfarlane and Murphy, 2010) but frequently display species- and tissue-specific expression patterns (Jopling, 2012; Sun et al., 2014). Regulatory changes by miRNA affect gene expression and can contribute to the emergence of new traits and phenotypic diversity (Yan et al., 2013; Li et al., 2020; Hao et al., 2023).

Postnatal phenotype is, to a large extent, determined during fetal growth and development. For example, myogenesis and adipogenesis are crucial developmental processes that begin *in utero* and have far-reaching effects on the manifestation of postnatal traits such as muscle mass, as the number of muscle fibres is determined before birth (Picard et al., 2002) and fat deposition, which plays a prominent role in the tenderness and taste of meat (Du et al., 2015; Louveau et al., 2016). The liver is a key metabolic organ, and its role in energy metabolism (Lotto et al., 2023), tightly links it with many of the economically important traits like adipogenesis and myogenesis. Moreover, the fetal liver is responsible for allocating incoming energy into different developmental processes (Rui, 2014), like muscle growth and thus, any transcriptional differences observed may shed light on possible causes of phenotypic differences between the two breeds. Our previous studies in purebred and crossbred taurine and indicine fetuses have demonstrated extensive parent-of-origin effects on the fetal musculoskeletal system (Xiang et al., 2013; Xiang et al., 2014) and revealed non-mendelian parent-of-origin effects on gene expression profiles (Liu et al., 2021) that may in part be orchestrated by differences in miRNA expression. Here, we report differential miRNA expression by breed and parent-of-origin effects in the same fetal resource of purebred taurine Angus, indicine Brahman and reciprocal cross fetuses, explore correlations with mRNA expression, and identify potential biological pathways targeted by these miRNAs to shed light on molecular differences that contribute to the diverse taurine and indicine cattle phenotypes.

## Materials and methods

### Study animals and sample collection

Liver samples of concepti used in the present study were the same as those described in our previous work (Liu et al., 2021). All animal experiments and procedures described in this study complied with Australian guidelines, approved by the University of Adelaide’s Animal Ethics Committee and followed the ARRIVE Guidelines (https://arriveguidelines.org/) (Approval No. S-094-2005). Experimental concepti and samples were obtained as described in Xiang et al. (2013) and Liu et al. (2021). Briefly, the parents were purebred Angus (*Bos taurus taurus*) and purebred Brahman (*Bos taurus indicus*), denoted as BT and BI, respectively. Primiparous dams of ∼16–20 months of age were grazed on pasture supplemented with silage. The animals were inseminated with purebred Angus (BTBT) or purebred Brahman (BIBI) semen and pregnancy tested via ultrasound. Pregnant dams and fetuses were ethically sacrificed at day 153 ± 1 of gestation and fetuses were dissected to obtain liver tissue samples from the *Lobus hepatis sinister*. Samples were snap-frozen in liquid nitrogen and stored at −80°C until further use. The liver samples represented three female and three male individuals from each of the four genetic combinations: BT × BT, BT × BI, BI × BT, and BI × BI. The four genetic groups were denoted with the paternal breed listed first. For example, BIBT represents a fetus whose sire was Brahman (BI) and whose dam was Angus (BT).

### microRNA extraction and sequencing

Total RNA was extracted from fetal liver tissue using Qiagen RNeasy^®^ Plus Universal kit according to the manufacturer’s instruction. The microRNA libraries were made by using the Bioo Scientific^®^ NEXTflex™ Small RNA-Seq kit v3 and sequenced at the Australian Cancer Research Foundation, Adelaide, Australia, using an Illumina^®^ NextSeq 500. Individual sample names and their corresponding genetic group are outlined in Supplementary Table S1.

### Sequence alignment

MicroRNA sequences were aligned using a custom Nextflow pipeline (Di Tommaso et al., 2017). Sequencing reads were first checked for quality using FastQC (v. 0.12.1) (Andrews, 2010). Adapters from the 5′ and 3′ ends of the reads were then trimmed using CutAdapt (v. 4.3) (Martin, 2011). We then filtered reads to retain those within the 17-28 bp range with a mean sequence quality of 25 using Prinseq (v. 0.20.4) (Schmieder and Edwards, 2011). Reads passing the filtering were reassessed with FastQC. Reads were then filtered for various bovine small RNA species that included ribosomal RNA (rRNA), transfer RNA (tRNA), small nuclear RNA (snRNA), and small nucleolar RNA (snoRNA). These small RNAs were downloaded from the Rfam database (Kalvari et al., 2018; Kalvari et al., 2020) via RNA Central and accessed on the 28th of April 2023. Next, we filtered reads against non-coding RNA (ncRNA) and coding DNA (cDNA) from Ensembl release 109 for ARS-UCD1.2. Reads that did not map to these RNA species were considered potential miRNAs and used in the subsequent analyses. Code relating to all analyses is available at: https://github.com/DaviesCentreInformatics/MicroRNA_BiVsBt. MiRNA sequencing reads are available from BioProject: PRJNA626458.

Messenger RNA from RNA-seq reads of the same samples (BioProject PRJNA626458) were aligned to ARS-UCD1.2 (Rosen et al., 2020) using the parameters described in Liu et al. (2021). Reads were quantified using featureCounts from the Rsubread package (v. 2.14.2) (Liao et al., 2019) and the ARS-UCD1.2 Ensembl gene annotation version 109.

### Quantification of known and discovery of novel miRNAs

Potential miRNA reads were used as input for the miRDeep2 pipeline (Friedländer et al., 2011). We first used “mapper.pl” from miRDeep2 with default parameters, except “-l,” which we set to 17. This step produced collapsed reads and alignments in the miRDeep2 “arf” format, which were then used in the miRDeep2 quantification and discovery steps. As input to “miRDeep2.pl,” we used the collapsed reads from the “mapper.pl” step, the ARS-UCD1.2 reference genome, the “.arf” file from the “mapper.pl” step, mature and hairpin miRNAs belonging to *Bos taurus*, which is denoted as bta from miRbase (Kozomara and Griffiths-Jones, 2011). We also used mature miRNAs from *Capra hircus* (chi) and *Ovis ares* (oar) in the miRDeep2 pipeline. We used a mirDeep2 score of ≥4, an estimated probability of being a true positive ≥70%, a significant Randfold *p*-value and the precursor location as an ID to identify novel miRNAs (Mukiibi et al., 2020).

### Identification of differentially expressed miRNAs and mRNAs

We used the output from miRDeep2 to generate counts for differentially expressed miRNA (DEM) analysis. We followed a standard differential expression workflow using DESeq2 (Love et al., 2014). All miRNA samples were sequenced in a single batch. The model parameters were breed and sex, with the contrasts being the six pairwise comparisons of each breed and the comparison between males and females.

The mRNA counts were quantified using featureCounts, and the output was used as input to DESeq2 for differential expression analyses (Love et al., 2014). Here, the model parameters were breed, sex and batch. The contrasts were the same as those used in the miRNA analyses. Only DEGs and DEMs with an adjusted *p*-value less than 0.05 were considered significant.

### miRNA target prediction

The miRanda (v. 3.3a) (Enright et al., 2003) miRNA target prediction software was used to identify possible gene targets of miRNAs. We extracted the three prime untranslated regions (3′UTRs) from the ARS-UCD1.2 Ensembl release 109. We then extracted the sequences of all mature miRNAs of interest, e.g., DEMs and group-specific miRNAs. We then aligned mature miRNA sequences to the 3′UTRs of ARS-UCD1.2 using miRanda with default parameters (Enright et al., 2003).

### miRNA and mRNA co-expression

The weighted gene co-expression network analysis (WGCNA) package (v. 1.72) was used to identify miRNA and mRNA co-expression networks (Langfelder and Horvath, 2008). WGCNA enables users to identify which genes have similar expression profiles (likely co-expressed) and to correlate that gene expression with other data like phenotype data or miRNA expression.

WGCNA removes genes with insufficient counts across samples, outlier genes, and outlier samples from the normalized count matrix. We used a variance stabilising transformation to normalise the matrix, as recommended by Langfelder and Horvath (2008). The input matrix is of the form *m* × *n* where *m* is the number of samples and n is the number of genes.

We then constructed the gene co-expression network by calculating an adjacency matrix from the filtered and normalized count matrix. The adjacency matrix is an *n* × *n* matrix, where *n* is the number of genes in the count matrix. The adjacency matrix is populated with values between 0 and 1, such that *a*_*ij*_ gives the connection strength between gene *i* and gene *j*. We used a “softPower” of five and seven in calculating the adjacency matrix for the parent of origin and breed comparisons, respectively. We then transformed the adjacency matrix into a topological overlap matrix (TOM) to calculate which genes have high topological overlap, i.e., are connected to roughly the same genes as one another. We next identified the dissimilarity TOM by subtracting the TOM from 1. Following this, we performed hierarchical clustering to identify genes that grouped into modules of co-expressed genes. Each module needed to contain at least 90 genes to be considered separate from another module, and modules needed a correlation of at least 0.8 to be merged. We then correlated the expression of these gene modules with each of the genetic groups and the corresponding miRNA expression data. We considered any correlation between miRNA, mRNA and genetic group with a *p*-value below 0.05 as significant. Significant modules were labelled first by a prefix denoting the comparison they were identified in and then with a letter. For example, module A, identified between the breeds, was labelled module br-A, module B, identified in the maternal comparison, was labelled module m-B, and module C, identified in the paternal comparison, was labelled module p-C.

### DEGs in miRNA targets

To determine if the predicted targets of a given miRNA were more likely to be DE within the present study, we tested how likely the targets of each miRNA were to be DE over all DE genes. Here, we defined the number of trials (*n*) as the number of predicted targets for a given miRNA, I.e., there were *n* targets that could be DE or not. The successes (*k*) were the number of predicted targets that were also DE. Finally, the probability of success (*p*) was the number of DEGs in a given comparison divided by the total number of genes in the count matrix. We paired the DEM and DEG comparisons such that if we were comparing DEMs identified between BIBI and BTBT, we only considered DEGs that were also identified between BIBI and BTBT.

### KEGG pathway analyses

The clusterProfiler R package (v. 4.8.1) (Yu et al., 2012) was used to perform KEGG pathway analysis. The “enrichKEGG” function was used to identify KEGG pathways significantly enriched in target genes. We used a cut-off of 0.05 for *p*-values, and the Benjamini-Hochberg method was used to adjust *p*-values to give q-values, where we also used a cut-off of 0.05 for significance testing. We performed KEGG analyses for the predicted targets of each miRNA that was either DE or specific to a particular group.

### Determination of most frequently targeted pathways

To determine which pathways were most frequently targeted by either DE or group-specific miRNAs, we first performed target prediction for each miRNA. Next, we performed KEGG pathway enrichment with clusterProfiler (v. 4.8.1) (Yu et al., 2012) for the genes targeted by each miRNA. We then identified all unique pathways and counted the number of times they were targeted by a different miRNA. Word clouds to represent frequency of pathways were made using wordcloud (v. 1.9.2. http://amueller.github.io/word_cloud/) against Angus and Brahman cattle outline as background.

## Results

### miRNA and mRNA sequence quality

**FIGURE 1:**
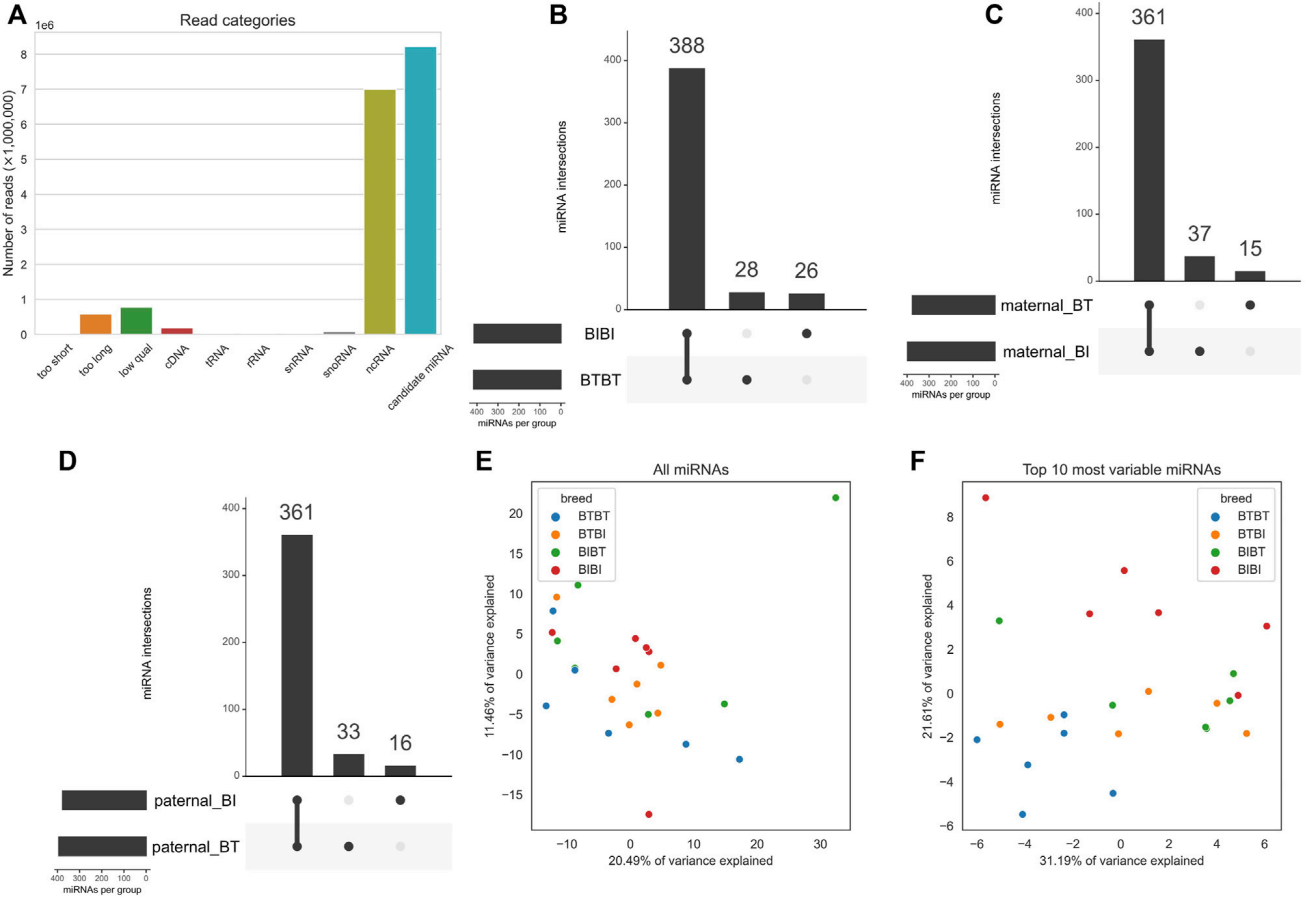
**(A)** Bar chart showing mean proportions of sequence reads belonging to the filtered, cDNA, tRNA, ncRNA, rRNA, snoRNA, and snRNA, too short, too long and “low qual” categories. Filtered refers to reads that are not contaminants and passed length and quality thresholds. cDNA, tRNA, ncRNA, snoRNA, and snRNA refer to any reads that mapped to them, i.e., contaminants. **(B)** Upset plot outlining the intersections of miRNAs with a count of at least one in all samples within a group. The x-axis of the bar plot represents each combination of groups e.g., the first point refers to the group of miRNAs found in all groups. The second point refers to the miRNAs found in BIBI, BTBT and BTBI but not BIBT. BT denotes Angus, and BI denotes Brahman. The y-axis is the number of miRNAs found in each point on the x-axis. **(C)** PCA plot showing how all 24 samples cluster when using all expressed miRNAs. Samples are coloured by breed, where blue denotes BTBT, orange is BTBI, green is BIBT and red is BIBI. **(D)** Same as **C** but only using the top ten most variable miRNAs determined by variance. The colours are the same as in **(C)**. **(E)** PCA plot showing clustering of samples using all expressed miRNAs. Dots represent an individual, and colours represent genetics. Blue dots represent BTBT, orange dots represent BTBI, green dots represent BIBT, and red dots represent BIBI. The x-axis represents PC1, and the y-axis represents PC2. **(F)** PCA plot showing clustering of samples using only the top ten most variable miRNAs based on variance. Colours and axes are the same as in **(E)**.

There was a sample average of ∼16.9 million reads before filtering (Supplementary Table S2). No reads were shorter than our minimum length threshold, but around 588,000 reads per sample were longer than our maximum length threshold, and around 779,000 reads were below our quality threshold and thus removed. This initial filtering left ∼15.5 million reads to be assessed as potential miRNAs. Just over 50% of reads were found to be other RNA species, not miRNAs (Figure 1A). Most non-miRNA RNA species were non-coding RNA (ncRNA), with an average of 7 million reads per sample mapped to known bovine ncRNA sequences (Figure 1A; Supplementary Table S2). After filtering, ∼8.2 million candidate miRNA reads remained for downstream analyses. Our previous work has reported the RNA-seq alignment quality in detail (Liu et al., 2021). Briefly, after read trimming, an average of 49.9 million reads remained for alignment, and between 72% and 77% of them could be assigned to genes.

### Ten miRNAs can differentiate taurine and indicine breeds

We performed principal component analyses to determine if the samples clustered by genetic group using the miRNA data as they did with RNA-seq data (Liu et al., 2021). We observed no discernible clustering pattern when using all miRNAs (Figure 1E). We then filtered the miRNA data and used the ten most variable miRNAs, as measured by variance. We subsequently observed a clear demarcation between BTBT and BIBI samples (Figure 1F). The hybrid groups (BIBT; BTBI) showed considerable overlap with one another and the purebred samples. The BTBT samples tended to cluster more closely to other samples within their group than any other group with BIBT, BTBI, and BIBI samples spanning the full range of PC1 (Figure 1F).

### Known miRNA expression and novel miRNA profiles

As the hybrid samples showed high similarity with both BIBI and BTBT genetic groups, we focused known miRNA expression on the purebred genetic groups. There were 414 and 416 known miRNAs with a normalized count of at least one in all BIBI and BTBT samples, respectively (Figure 1B; Supplementary Table S3). Most (388) known miRNAs were shared between BIBI and BTBT samples (Figure 1B). In the BTBT group, we found 28 miRNAs not expressed in the BIBI group. Conversely, we found 26 miRNAs in the BIBI group not expressed in the BTBT group (Figure 1B). In 21 of the 24 liver samples analysed, bta-miR-122 was the most highly expressed miRNA, with an average of 39% of all mapped reads. This liver-specific miRNA is conserved across species with pleiotropic functions, including in cholesterol, glucose and iron homeostasis and lipid metabolism (Thakral and Ghoshal, 2015).

When we separated fetal liver samples based on the breed of their maternal and paternal parents, there were between 376 and 398 miRNAs expressed in all 12 samples for each parental group, i.e., maternal BI, maternal BT, paternal BI and paternal BT (Supplementary Table S3; Figures 1C, D). We observed 361 miRNAs shared between the maternal BT and BI groups; a similar number was observed when we compared paternal BI and BT groups. The maternal BI group samples contained 37 miRNAs not expressed in the maternal BT group, while 15 miRNAs were unique to the maternal BT group when compared to the maternal BI group (Figure 1B). The inverse of this pattern was observed between paternal groups, where samples from the paternal BT group had 33 unique miRNAs compared to 16 in those from the paternal BI group (Figure 1C).

We discovered eight potentially novel miRNAs not listed in miRBase in BTBT and BIBI samples (Supplementary Table S4). We considered these high-confidence novel miRNAs as they were found in all samples within a particular group. Six of these novel miRNAs were shared between BIBI and BTBT samples, with one being exclusive to each group, i.e., one novel miRNA was found in all six BTBT samples but not BIBI samples and *vice versa* (Supplementary Table S4). We have included the sequences of these novel miRNAs in Supplementary Table S4. Given all predicted novel miRNAs were lowly abundant (less than 0.2% of filtered reads) and these are not validated, we did not analyse them further.

### Gene expression modules correlated with breed

**FIGURE 2:**
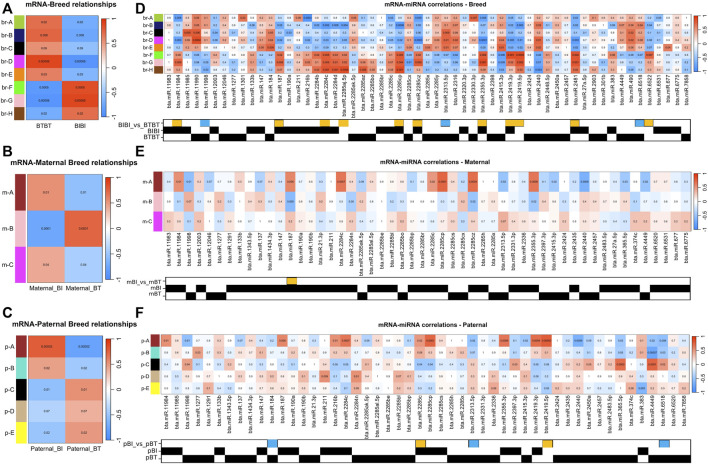
**(A)** Correlation heat map displaying the correlations between traits and the mRNA expression data. The x-axis refers to each phenotypic trait, i.e., breed. The y-axis refers to each of the gene modules identified by WGCNA. Each cell in the heatmap is coloured by the strength of the correlation, where a darker shade of red denotes an increasingly positive correlation, and darker shades of blue denote an increasingly negative correlation. The values in the heatmap denote the *p*-value associated with the correlation. **(B)** Same as **A** but comparing maternal genetics. **(C)** Same as **(A,B)** but comparing paternal genetics. **(D)** Correlation heat map displaying the correlations between mRNA and miRNA. The x-axis denotes each of the miRNAs deemed of interest, i.e., was differentially expressed or only found in a particular group. The y-axis and correlation colour scheme are the same as **(A)**. The matrix underneath the heatmap denotes which comparison or group within which a given miRNA was identified. A yellow box denotes a miRNA that was upregulated in BIBI, and a blue box denotes a miRNA that was upregulated in BTBT. BT denotes Angus, and BI denotes Brahman. A black square denotes the presence of that miRNA in that group. **(E)** Same as **(D)** but comparing miRNAs expressed in samples with different maternal genetics. A yellow box denotes miRNA that was upregulated in maternal BI. **(F)** Same as **(D,E)** but comparing samples with different paternal genetics. Yellow boxes denote miRNAs upregulated in paternal BI, and blue boxes denote miRNAs upregulated in paternal BT.

Using the WGCNA method (Langfelder and Horvath, 2008) and a significant correlation threshold of *p* < 0.05, we identified seven gene modules with significant correlations and one approaching (*p* = 0.09) our significance threshold (*p* < 0.05) among BIBI and BTBT groups (Figure 2A). These modules, A-H, comprised a total of 7,707 genes, with 239 of these being differentially expressed between BIBI and BTBT samples. We identified a total of 2,525 DEGs between BIBI and BTBT (Table 1), and together, these eight modules contained 239 of 2,525 (∼9%) DEGs identified between the two groups. Modules A, B, D and E displayed significant positive correlations with BTBT samples and significant negative correlations with BIBI samples. Modules F, G and H displayed significant negative correlations with BTBT samples and significant positive correlations with BIBI samples. Module C had a positive trend with BTBT samples and a negative trend with BIBI samples but was not statistically significant (*p* = 0.09).

**TABLE 1:** Number of differentially expressed miRNAs and genes of breed and parent-of-origin effects.

	BIBI—BTBT	Maternal BI—Maternal BT	Paternal BI—Paternal BT
*miRNA*			
Up	11	1	2
Not significant	910	923	919
Down	3	0	3
**Total DE**	**14**	**1**	**5**
*mRNA*			
Up	1,290	279	1,030
Not significant	16,128	18,018	16,556
Down	1,235	356	1,067
**Total DE**	**2,525**	**635**	**2,097**

The reference groups for the three comparisons are BTBT, Maternal BT and Paternal BT.

BTBT denotes purebred Angus samples. i.e., Angus (BT) sire and Angus (BT) dam.

BIBI denotes purebred Brahman samples. i.e., Brahman (BI) sire and Brahman (BI) dam.

Maternal BI denotes all samples with a Brahman dam (BTBI; BIBI).

Maternal BT denotes all samples with an Angus dam (BIBT; BTBT).

Paternal BI denotes all samples with a Brahman sire (BIBT; BIBI).

Paternal BT denotes all samples with an Angus sire (BTBI; BTBT).

Bold values denote the total of the up and down regulated miRNAs and mRNAs. For example in the BIBI—BTBT comparison, 11 miRNAs were upregulated and 3 were down regulated = 14.

We then performed KEGG pathway analysis on the genes within each module to determine if they were enriched in pathways that shed light on phenotypic differences between Brahman and Angus. Among the eight gene modules correlated with breed, four modules were enriched with pathways. Module D from the breed comparison (br-D) was enriched in eight pathways: amino sugar and nucleotide sugar metabolism, biosynthesis of nucleotide sugars, apoptosis, MAPK signalling, phosphatidylinositol signalling, one carbon pool by folate and lipid and atherosclerosis pathways. Module br-F displayed a single enrichment in the glutamatergic synapse pathway. Module br-G had enrichments in relaxin signalling, chemical carcinogenesis—reactive oxygen species, nicotinate and nicotinamide metabolism, thermogenesis, retrograde endocannabinoid signalling, GnRH signalling and purine metabolism pathways. Module br-C, while not significant, did approach a significant correlation with breed (*p* = 0.09) and displayed an enrichment of 32 pathways, including thermogenesis, TGF-beta signalling, mTOR signalling, neurotrophin signalling and Ras signalling (Supplementary Table S5). Modules br-A, br-B, br-E, and br-H were not enriched in any pathways.

### Gene expression modules correlated with maternal genome

Three gene modules identified in the maternal comparison (m-A, m-B, and m-C) significantly correlated with maternal BI or BT groups (Figure 2B). These modules contained 214 DEGs, which was about 34% of the total differentially expressed genes (635) identified between maternal breed groups (Table 1). Modules m-A and m-C were positively correlated with the maternal BI group and negatively correlated with the maternal BT group. Module m-B was negatively correlated with the maternal BI group and positively correlated with the maternal BT group (Figure 2B). All three modules correlated with the maternal breed were enriched in pathways (Supplementary Table S5). Module m-A showed enrichment for the glutamatergic synapse and several other pathways (Supplementary Table S5). Pathways enriched in module m-B included ECM-receptor interaction and PI3K-Akt signalling pathways, among others (Supplementary Table S5). Module m-C displayed enrichments in cell cycle and DNA replication pathways, as well as several others (Supplementary Table S5).

### Gene expression modules correlated with paternal genome

Using WGCNA, we identified four gene modules with significant correlations and one module approaching significance (*p* = 0.07) with paternal groups (Figure 2C). There were 13,805 genes in these modules, with 1,903 differentially expressed, i.e., ∼65% of the 2,097 DEGs identified between paternal groups were contained within these five gene modules. Modules A and B identified in the paternal group comparison (p-A, p-B) were positively correlated with paternal BI samples and negatively correlated with paternal BT samples. The remaining modules (pC-E) were negatively correlated with paternal BI and positively correlated with paternal BT samples (Figure 2C).

All five modules correlated with paternal breeds were enriched in pathways (Supplementary Table S5). Purine metabolism, bile secretion, glutamatergic synapse and pancreatic secretion were all enriched in module p-A. Module p-B was enriched in 84 pathways, too many to list; refer to (Supplementary Table S5). Module p-C saw, among others, an enrichment in MAPK signalling and CoA biosynthesis pathways (Supplementary Table S5). Module p-D was enriched for the spliceosome pathway. Module p-E was enriched for 20 pathways, including thermogenesis and purine metabolism.

### miRNA-module correlations

There were 78 miRNAs identified as being either differentially expressed or only expressed in a breed, maternal breed or paternal breed group. Of these, 35 had a significant positive correlation with at least one module, and 27 had a significant negative correlation with at least one module (Supplementary Table S7). The greatest number of modules that were significantly correlated with a single miRNA was four. Bta-miR-2284c was positively correlated with modules br-F, br-G, br-H, m-A, and p-A and negatively correlated with br-B, br-D (Figures 2D–F; Table 2). Bta-miR-2285cp was positively correlated with br-F, br-G, br-H, m-A, and p-A, and negatively correlated with br-B, br-D and p-C (Figures 2D–F; Table 2).

**TABLE 2:** Positive and negative correlations between miRNAs and gene expression modules.

*miRNA*	*Positively correlated module*	*Negatively correlated module*	*Differentially expressed*	*Group-specific*
bta-miR-11984	br-G, m-A, p-A	br-A, br-D	BIBI	BIBI maternal, BIBI paternal
bta-miR-11985	br-C	-	-	BIBI, BIBI paternal
bta-miR-11990	br-C, br-D	-	-	BTBT
bta-miR-11998	p-C	br-H, br-G, m-A, p-A	BTBT	BTBT maternal, BTBT paternal
bta-miR-12003	-	br-C	-	BTBT
bta-miR-1277	p-B	-	-	BTBT paternal
bta-miR-1301	br-A	-	-	BTBT
bta-miR-184	br-D, br-E	-	-	BTBT
bta-miR-187	br-F, br-G, m-A, p-A	br-B, br-D, m-B	BIBI, BIBI maternal	BIBI paternal
bta-miR-190a	-	br-C	-	BIBI
bta-miR-211	p-D		-	BIBI paternal
bta-miR-216b	br-G, p-A	br-A, br-D	-	BIBI, BIBI paternal
bta-miR-2284b	br-F, br-H	br-B	-	BIBI
bta-miR-2284c	br-F, br-G, br-H, m-A, p-A	br-B, br-D	BIBI	BIBI maternal, BIBI paternal
bta-miR-2284d	br-F, br-G, br-H	br-B	-	BIBI
bta-miR-2284n	p-D	-	-	BTBT paternal
bta-miR-2285aj-5p	br-F, br-G, br-H	br-B	BIBI	-
bta-miR-2285az	br-H	-	-	BTBT
bta-miR-2285c	m-A, p-A	br-A,p-C	BIBI paternal	BIBI, BIBI maternal
bta-miR-2285cp	br-F, br-G, br-H, m-A, p-A	br-B, br-D, p-C	BIBI	BIBI maternal, BIBI paternal
bta-miR-2285cx		br-G	-	BTBT
bta-miR-2285cz	br-F, br-G, m-A	br-A, br-D, m-B	BIBI	BIBI maternal
bta-miR-2313-5p	br-C, br-D, br-E	m-A, p-A	BTBT	BTBT maternal, BTBT paternal
bta-miR-2316	br-D	br-F	-	BTBT
bta-miR-2330-3p	br-A	-	-	BTBT
bta-miR-2355-3p	br-F, br-G, m-A, p-A	br-A, br-D, m-B	BIBI	BIBI maternal, BIBI paternal
bta-miR-2397-3p	p-C	-	-	BIBI paternal
bta-miR-2415-3p	br-D,br-E	br-F, br-G, br-H,m-A, p-A	-	BTBT, BTBT maternal, BTBT paternal
bta-miR-2419-3p	br-F, br-G, p-A	br-B, br-D, br-E	BIBI	BIBI paternal
bta-miR-2419-5p	br-F, br-G, br-H, p-A	br-B, br-D, br-E	BIBI	BIBI paternal
bta-miR-2440	br-B, br-D, br-E	br-F, br-G, br-H, m-A, p-A	-	BTBT, BTBT maternal, BTBT paternal
bta-miR-2481	br-F, br-G, br-H	br-A, br-B, br-D, br-E	BIBI	-
bta-miR-365-5p	p-C	m-A, p-A	-	BIBI maternal, BTBT paternal
bta-miR-383	-	p-E	-	BIBI paternal
bta-miR-4449	p-C	m-A, p-A, p-B	-	BTBT maternal, BTBT paternal
bta-miR-490	br-C, br-D	-	-	BTBT
bta-miR-6518	br-B, p-C	p-A, p-B	BTBT, BTBT paternal	-
bta-miR-6522	br-F	br-B	BIBI	-
bta-miR-677	br-E	-	-	BIBI

The “Differentially expressed” column denotes if that miRNA was differentially expressed. A value of “BIBI” in the “Differentially expressed” column for a given miRNA means that miRNA was differentially expressed between breed BTBT and BIBI and was upregulated in BIBI. Similarly, a value of “BTBT paternal” in the “Differentially expressed” column means that miRNA was differentially expressed between paternal BIBI and paternal BTBT groups and was upregulated in the paternal BTBT group. The “Group-specific” column refers to miRNAs that were only expressed in the given group.

BTBT denotes purebred Angus samples. I.e., Angus (BT) sire and Angus (BT) dam.

BIBI denotes purebred Brahman samples. I.e., Brahman (BI) sire and Brahman (BI) dam.

Maternal BI denotes all samples with a Brahman dam (BTBI; BIBI).

Maternal BT denotes all samples with an Angus dam (BIBT; BTBT).

Paternal BI denotes all samples with a Brahman sire (BIBT; BIBI).

Paternal BT denotes all samples with an Angus sire (BTBI; BTBT).

The “br” prefix before the module letter ID, e.g., br-A, denotes module A identified between BIBI and BTBT samples.

The “m” prefix before the module letter ID, e.g., m-A, denotes module A identified between maternal BI and maternal BT samples.

The “p” prefix before the module letter ID, e.g., p-A, denotes module A identified between paternal BI and paternal BT samples.

Bta-miR-2415-3p was positively correlated with br-D and br-E, while it also exhibited negative correlations with br-F, br-G, br-H, m-A, and p-A (Figures 2D–F; Table 2). Bta-miR-2440 was positively correlated with br-B, br-D and br-E and was negatively correlated with br-F, br-G, br-H, m-A, and p-A (Figures 2D–F; Table 2). Interestingly, bta-miR-2284c and bta-miR-2285cp had very similar module correlation patterns, the only difference being that bta-miR-2285cp was also negatively correlated with module p-C. Similarly, bta-miR-2415-3p and bta-miR-2440 had similar module correlation patterns, the only difference being that bta-miR-2440 was also positively correlated with module br-B. Furthermore, bta-miR-2284c and bta-miR-2285cp were positively correlated with the BIBI, maternal BIBI and paternal BIBI groups and displayed expression below our significance threshold in BTBT samples (Figures 2D–F).

### Predicted targets of DEMs more likely to be DEGs

We compared the predicted targets of each DEM identified between BIBI and BTBT samples and determined whether they were more likely to be DE than the background genes, i.e., genes not predicted to be targeted by a given miRNA. We found that predicted targets of 12 of the 14 DEMs identified between BIBI and BTBT samples were significantly more likely to be DE than non-target genes (binomial test, *p* < 0.05) (Supplementary Table S8). Furthermore, the targets of the single DEM identified between maternal BI and maternal BT were significantly more likely to be DE (binomial test, *p* < 0.05). The targets of four of the five DEMs identified between paternal groups were significantly more likely to be DE than non-target genes (binomial test, *p* < 0.05).

### Pathways predicted to be targeted by DE and group-specific miRNAs

**FIGURE 3:**
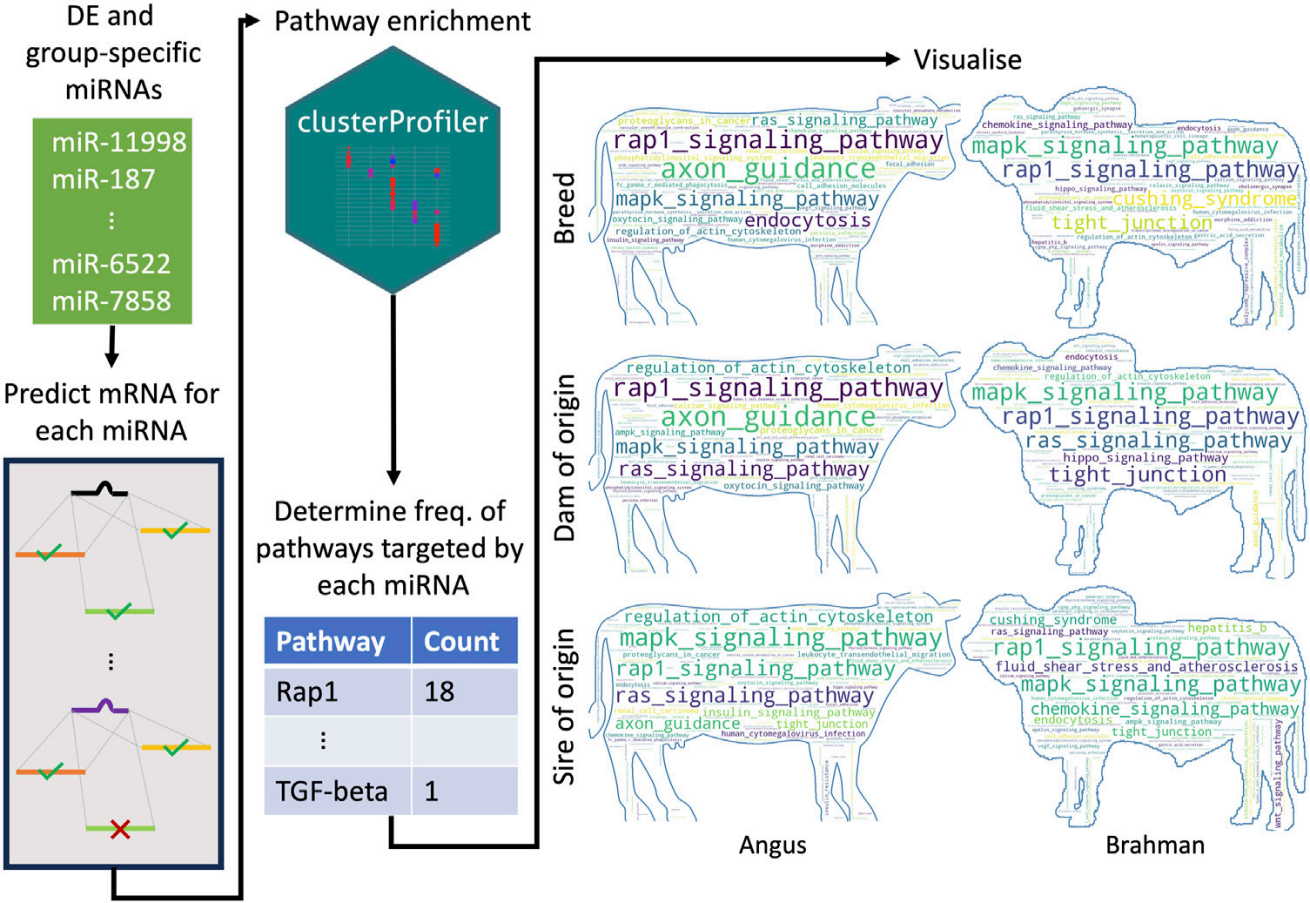
Schematic overview of how the most frequently targeted pathways were identified. For each differentially expressed and group-specific miRNA, we predicted their mRNA targets. We then performed pathway enrichment, where the input was a list of genes predicted to be targeted by a given miRNA. We then identified all the unique pathways predicted to be targeted by all the upregulated and expressed miRNAs for a particular group (e.g., all upregulated and expressed miRNAs in the BTBT group) and counted how many miRNAs targeted each pathway. BT denotes Angus, and BI denotes Brahman. The number of miRNAs upregulated and expressed in each group was BIBI = 29, BTBT = 28, maternal BI = 37, maternal BT = 15, paternal BI = 17 and paternal BT = 33.

There were 23, 15, 33, 29, 37, and 17 miRNAs upregulated or only expressed in breed BTBT, maternal BT, paternal BT, breed BIBI, maternal BI and paternal BI, respectively (Figure 3A; Supplementary Table S9). We used the miRanda target prediction software (v. 3.3a) (Enright et al., 2003) to identify potential targets of each miRNA (Figure 3). Between 300 and 5,115 targets were predicted among these miRNAs (Supplementary Table S10), which were then used to perform KEGG pathway analysis. Genes involved in the MAPK and Rap1 signalling pathways were consistently targeted by miRNAs, with between 37% and 56% of miRNAs in each group targeting these pathways (Supplementary Table S9). All pathways targeted by miRNAs expressed in each group can be seen in Figure 3; Supplementary Table S9.

### No DEMs were identified between the sexes

To determine potential differences in miRNA expression between the sexes, we compared all males against all females. We observed no DEMs, nor did we find significant correlations between gene modules and sex. Consequently, there was no pathway analysis performed. The lack of DEMs between males and females was consistent with the limited number of DEGs observed between male and female samples.

## Discussion

To our knowledge, this study is the first to report on miRNA expression differences between taurine and indicine cattle and correlate the results with mRNA expression. We utilized miRNA expression data from fetal liver samples representing male and female individuals of Brahman, Angus and their reciprocal crosses. The inclusion of reciprocal crosses enabled us to disentangle not only miRNA expression differences that could be related to breed but also whether the maternal or paternal genomes impacted miRNA expression. Previous efforts to understand potential miRNA drivers of differences between the two cattle subspecies have focused on adult tissue. For example, Deb and Sengar (2021) investigated the miRNA expression profiles between Sahiwal (indicine) and Frieswal (indicine x taurine) cattle from blood samples in response to summer heat stress, identifying DEMs that interact with heat shock protein 70 (*Hsp*70). Similarly, Dong et al. (2023) investigated miRNA expression differences in the mature testes of Mongolian (taurine) and Hainan (indicine) cattle, demonstrating breed differences in spermatogenesis-related miRNAs. However, it is reasonable to assume that molecular drivers of physiological and phenotypic differences may not be detectable by the time the trait is fully developed in adults.

Our previous study on mRNA expression data in the same fetal resource clearly distinguished genetic groups BTBT, BIBT, BTBI, and BIBI (Liu et al., 2021). In contrast, miRNA data only separated purebred taurine and indicine fetal liver samples. Furthermore, despite observing thousands of differentially expressed genes between BTBT and BIBI samples, we identified only 14 DEMs between the two breeds. This result is similar to a study that observed only 23 DEMs when comparing miRNA expression in Hereford x Limousine beef cattle and Holstein-Friesian dairy cattle muscle cells during myogenic differentiation (Sadkowski et al., 2018). As a single miRNA targets multiple mRNAs (Peterson et al., 2014), the relatively low number of DEMs is not unexpected as these miRNAs may profoundly impact genes and gene networks differentially expressed between breeds.

Using the mRNA expression data, we identified gene modules correlated with the two breeds, Angus and Brahman, that represent taurine and indicine cattle. Moreover, by correlating the same gene expression data with miRNA expression, we identified miRNAs that may contribute to the gene expression differences that distinguish the two types of cattle. For example, module br-C was positively correlated with BTBT samples and was enriched for several pathways, including thermogenesis and Ras signalling. Ras signalling plays an essential role in adipogenesis (Murholm et al., 2010); adipocyte hyperplasia is evident in cattle at the mid-gestation developmental stage (Zhao et al., 2019). Interestingly, bta-miR-11990 was predicted to target genes involved in the Ras signalling pathway. This miRNA shares a similar correlation pattern with several BTBT-specific miRNAs (bta-miR-2313-5p, bta-miR-490, and bta-miR-2316), which were predicted to target pathways also involved in adipogenesis, such as NF-kappa B signalling (Peng et al., 2022) and VEGF signalling pathways (Park et al., 2017). Moreover, bta-miR-2316 has previously been reported as being expressed in the adipose tissue of cattle, suggesting a role in adipogenesis (Romao et al., 2014). However, further work is needed to confirm its role in adipogenesis in cattle. As Angus cattle are known to have superior fat deposition performance in cold climates compared to Brahman (Boyles and Riley, 1991), these miRNAs may be contributing to post-transcriptional regulation that conveys this trait to Angus cattle. However, the potential correlation of these miRNAs with fat deposition needs to be tested experimentally.

An advantage of including reciprocal crosses in breed comparisons is that it enables the investigation of maternal and paternal genome effects. We observed a single DEM between different maternal groups. In addition to this single DEM (bta-miR-187), we observed several miRNAs that were only expressed in maternal BI samples (bta-miR-2284c, bta-miR-2285c, bta-miR-2285cp, bta-miR-2285cz, bta-miR-2355-3p, and bta-miR-11984). These miRNAs were positively correlated with module m-A, a module that was significantly correlated with maternal BI samples and was enriched for the glutamatergic synapse pathway. Moreover, bta-miR-2355-3p was also predicted to target genes involved in this pathway. We observed a similar pattern of module-pathway associations in the sire of origin comparison, with bta-miR-2355-3p being positively correlated to module p-A, which was also enriched for the glutamatergic synapse pathway. Several miRNAs (bta-miR-187, bta-miR-216b, bta-miR-2284c, bta-miR-2285c, bta-miR-2285cp, bta-miR-2419-3p, bta-miR-2419-5p, and bta-miR-11984) shared a similar correlation pattern to bta-miR-2355-3p. The glutamatergic synapse has a known role in heat tolerance of an individual as glutamatergic neurons transmit peripheral and central heat signals to the hypothalamic preoptic area of the brain (Sun et al., 2022), which then begins a coordinated response to lower the temperature. The liver is known to play an integral role in coordinating this heat stress response via increased production of heat shock proteins, increasing metabolic rate and increased vasodilation (Thorne et al., 2020).

Further evidence to support the possible role of glutamatergic synapses in conveying heat tolerance in cattle can be found in another recent study of Holstein dairy cattle (Cheruiyot et al., 2021). Given the developmental timeline of the liver and the observed pathway enrichments (Tiniakos et al., 1996; Giancotti et al., 2019), it is possible that bta-miR-2355-3p and those miRNAs with similar correlation patterns play a role in modulating neuron development in the liver, priming it for hotter temperatures later in life and that this can be conveyed by either a Brahman sire or dam.

While we observed several DEMs that were correlated to gene modules, many more miRNAs were only expressed in one of the two groups in each comparison, e.g., bta-miR-490 in the breed comparison, bta-miR-11998 in the maternal breed comparison and bta-miR-187 in the paternal breed comparison. There was some overlap between DEMs and group-specific miRNAs, e.g., bta-miR-184 in the paternal-breed comparison. However, this was due to differences in the detection of expression threshold used to identify miRNAs as being expressed and how DESeq2 determines if a gene or miRNA should be retained. In any case, more miRNAs presented as only being expressed in one group, e.g., BIBI or BTBT, than displaying differential expression between groups. This pattern leads us to posit that group-specific miRNAs may be more important in driving gene regulatory differences than DEMs between Brahman and Angus cattle.

We identified a range of predicted targets for each of the differentially expressed and group-specific miRNAs and performed biological pathway analyses on these targets to gain insights into the pathways that may be affected. Notably, a substantial proportion of the targeted pathways identified in each of the three comparisons were signalling pathways, such as the Rap1, MAPK, and Ras signalling pathways. Each of these pathways plays an important role in fetal development. Rap1 signalling is important for vascular morphogenesis (Chrzanowska-Wodnicka, 2013). Additionally, the Rap1 signalling pathway has been implicated as a possible cause of the differences in meat production traits between high and low-performing meat goat breeds (Shen et al., 2022). Furthermore, Rap1 signalling ablation in the brain of mice has been shown to protect mice from high-fat diet-induced obesity (Kaneko et al., 2016), suggesting a critical role of Rap1 signalling in fat deposition. While this study investigated Rap1 signalling in the brain, given the close interactions between the brain and liver to monitor glucose and lipid homeostasis, it is possible that Rap1 signalling in the liver also has a role in fat storage. Ras and MAPK signalling pathways are critical to cell proliferation and differentiation (Zhang and Liu, 2002). Moreover, the Ras signalling pathway is purported to have a role in adipogenesis as ectopic expression of the pathway can induce preadipocyte formation in the absence of insulin and insulin-like growth factor 1 (*Igf-1*) (MacDougald and Lane, 1995), suggesting a possible role in modulating adipogenesis in the fetus. In addition, MAPK signalling can act as a negative regulator of muscle development (Xie et al., 2018), suggesting that differences in how this pathway is regulated may influence how the fetus develops muscle. The differences in miRNA expression between taurine and indicine cattle appear to result in differential modulation of signalling cascades involved in fetal liver development and growth regulation, which may contribute to phenotypic differences in traits such as heat tolerance, fat deposition and growth rate. In conclusion, we have identified differentially expressed miRNAs in the rapidly growing fetus that are involved in the genetic and epigenetic architecture of a range of complex traits, including fat deposition and heat tolerance, that differentiate taurine and indicine cattle, which are vital for global food supply.

## Data Availability

The datasets presented in this study can be found in online repositories. The names of the repository/repositories and accession number(s) can be found in the article/Supplementary Material.
