# Supplementary material for: MicroRNA breed and parent-of-origin effects provide insights into biological pathways differentiating cattle subspecies in fetal liver
Source: Front Genet. 2023 Dec 13;14:1329939. doi: 10.3389/fgene.2023.1329939 (PMC10757722; doi:10.3389/fgene.2023.1329939)
Supplement: Supplementary file 11 [file DataSheet1.DOCX]

*MicroRNA breed and parent-of-origin effects provide insights into biological pathways differentiating cattle subspecies*

Callum MacPhillamy, Yan Ren, Tong Chen, Stefan Hiendleder, Wai Yee Low*

*** Correspondence:** Wai Yee Low: [wai.low@adelaide.edu.au](mailto:wai.low@adelaide.edu.au)

**Supplementary Material 1.**

Table outlining the sample information for all 24 samples used in this study.

**Supplementary Material 2.**

Spreadsheets detailing the alignment statistics for the miRNA and mRNA mapping to ARS-UCD1.2.

**Supplementary Material 3.**

Spreadsheet containing data used to generate the upset plots.

**Supplementary Table 4.**

Table containing the novel miRNAs, their counts in each group and their sequence.

**Supplementary Table 5.**

Table containing the results of the pathway analysis.

**Supplementary Table 6.**

Tables containing the differentially expressed miRNAs.

**Supplementary Table 7.**

An expanded table of all module-miRNA correlations.

**Supplementary Table 8.**

Table displaying the binomial test results.

**Supplementary Table 9.**

Tables containing the pathway counts for each of the pathways that were predicted to be targeted by a miRNA.

**Supplementary Table 10.**

Table outline each miRNA, the number of mRNAs it is predicted to target and a ‘;’ separated list of the genes predicted to be targeted by the miRNA
